# Central metabolic pathway modification to improve L-tryptophan production in *Escherichia coli*

**DOI:** 10.1080/21655979.2019.1592417

**Published:** 2019-04-12

**Authors:** Lihong Du, Zhen Zhang, Qingyang Xu, Ning Chen

**Affiliations:** College of Biotechnology, Tianjin University of Science and Technology, Tianjin, China

**Keywords:** L-tryptophan, *Escherichia coli*, metabolic engineering, central metabolic pathway, PEP

## Abstract

Tryptophan, an aromatic amino acid, has been widely used in food industry because it participates in the regulation of protein synthesis and metabolic network *in vivo*. In this study, we obtained a strain named TRP03 by enhancing the tryptophan synthesis pathway, which could accumulate tryptophan at approximately 35 g/L in a 5 L bioreactor. We then modified the central metabolic pathway of TRP03, to increase the supply of the precursor phosphoenolpyruvate (PEP), the genes related to PEP were modified. Furthermore, citric acid transport system and TCA were upregulated to effectively increase cell growth. We observed that strain TRP07 that could accumulate tryptophan at approximately 49 g/L with a yield of 0.186 g tryptophan/g glucose in a 5 L bioreactor. By-products such as glutamate and acetic acid were reduced to 0.8 g/L and 2.2 g/L, respectively.

## Introduction

Tryptophan, an aromatic amino acid [1], plays an important role in various biological activities in living beings including humans. Tryptophan products are used in food additives, medicine, and nutritional therapy [2–4]. Moreover, tryptophan is a precursor for synthesizing 5-hydroxytryptamine [5], coenzymes, phytohormones, and other substances, which can improve human sleep quality and relieve mental fatigue [6]. L-tryptophan is produced by extraction, chemical synthesis [7,8], enzymatic, and microbial fermentation methods [9]. Recently, with the development of biotechnology, it has become a mainstream trend to produce L-tryptophan by microbial fermentation with a lower production cost and controllable production mode [10].

**Figure 1.**
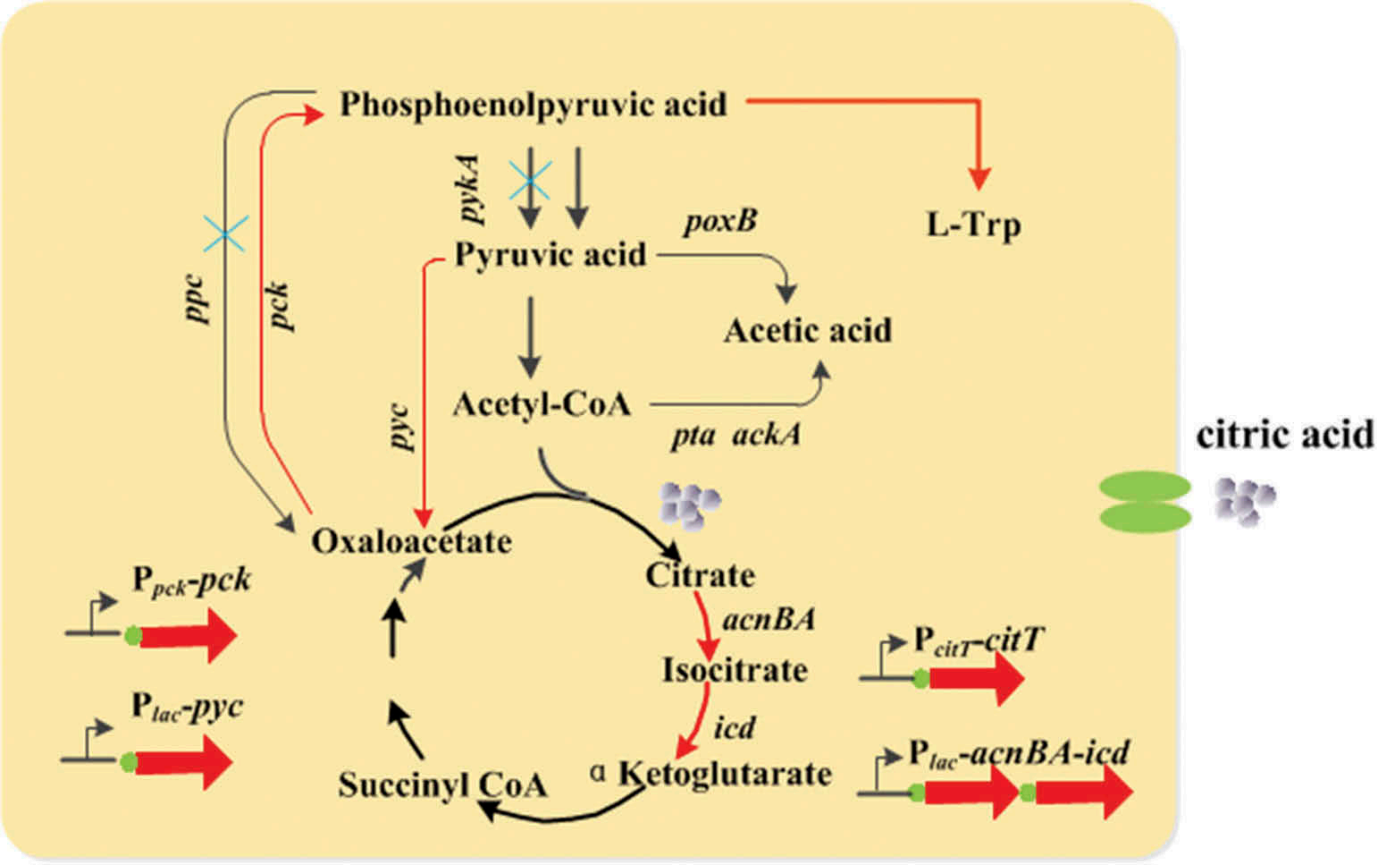
Central metabolic pathway in *E. coli* and the scheme for construction of tryptophan producing strains. Red arrows indicate overexpression of the relevant genes through chromosomal integration or promoter replacement. ‘X’ indicates deletion of the corresponding gene.

**Figure 2.**
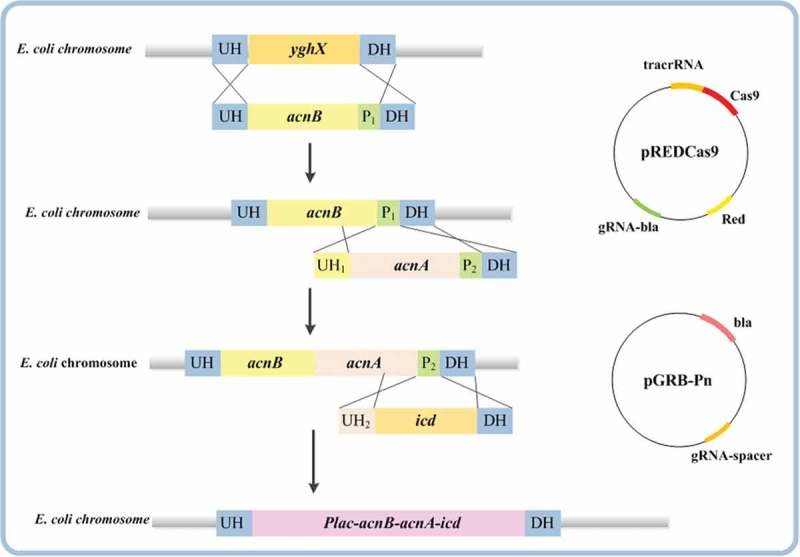
Strategy for multi-step integration and assembly of a large DNA fragment into *E. coli* chromosome. UH, UH1, and UH2 indicate the upstream homologous sequences, and DH indicates the downstream homologous sequence. P1 and P2 indicate the exogenous protospacer and PAMs from *S. pyogenes.*

Microorganisms commonly used in tryptophan production include *Escherichia coli* and *Corynebacterium glutamiens*, Although *Corynebacterium glutamatum* is a natural microorganism for producing amino acids [11], *E. coli* (*Escherichia coli*), which has a faster reproductive cycle and more mature genetic modification systems, has been widely applied to the microbial fermentation industry, including the production of amino acids, such as tryptophan, threonine [12], valine [13], tyrosine [14]; organic acids, such as malic acid [15], succinic acid [16], lactic acid [17], glutaric acid [18], and adipic acid [19], among others. In recent years, with the understanding of the biosynthetic pathways of terpenes and phenols, *E. coli* has been used to produce more complex compounds [20,21]. Besides, in view of the many advantages of *E. coli*, the production of tryptophan by *E. coli* has also been extensively studied.

In *E. coli*, tryptophan is metabolized [22] with the help of the glycolytic, pentose phosphate, shikimic acid, and chorismate pathways. In addition, various regulatory mechanisms such as feedback inhibition, feedback repression [23], and feedforward inhibition [24] synergistically regulate the synthesis of tryptophan. In order to accumulate tryptophan, the primary task involves relieving the feedback of the end products to key enzymes including 3-deoxy-d-arabino-heptulosonate-7-phosphate synthase and component I of anthranilate synthase [25,26]. When the microbial fermentation industry was in its initial stages of development, mutants that accumulate tryptophan were obtained using conventional mutagenesis techniques [27]. With the development of genome sequencing technology, positive feedback-resistant mutations were identified and applied to the construction of strains for tryptophan production. Integration of the *aroG* and *trpE* genes [28] with positive mutations into the *E. coli* genome could result in a significant increase in tryptophan accumulation. Deactivation of repressors, such as TrpR and TyrR, and modification of the transport pathways, such as Mtr, TnaB, and AroP, further increased tryptophan accumulation [29,30].It is also important to ensure a coordinated and balanced supply of precursors for the synthesis of tryptophan metabolism. PEP and erythrose-4-phosphate (E4P), as carbon skeleton precursors directly enter the shikimic acid pathway, are the most important precursors for tryptophan synthesis. The main function of PEP is to enter the TCA cycle via pyruvate to provide energy for cell growth, and the proportion of carbon metabolic flow into the shikimic acid pathway usually does not exceed 2% [31]. In addition, L-glutamine, L-serine, and PRPP also play different roles in tryptophan biosynthesis [32,33]. However, weakening the PEP-to-pyruvate pathway to enhance carbon flux of the tryptophan biosynthesis pathway affects cell growth [34]. Although many attempts have been made to produce tryptophan by microorganisms, the key to further improve tryptophan production is to further achieve the dynamic balance between biomass formation and tryptophan production.

In the study, we did a lot of experiments to optimize the balance between biomass formation and tryptophan production. Schematic map of tryptophan synthesis pathway modification was showed in Figure 1. First, TRP1was chosen as a starting strain, in order to preliminarily accumulate tryptophan, the degradation pathway of tryptophan was downregulated and the key genes of the shikimic acid and chorismate pathways were upregulated, the strain named TRP03 was obtained, and tryptophan could be accumulated at approximately 8.1 g/L in shake flask culture for 24 h. Next, TRP03 was selected as a basics strain to modify the EMP pathway and TCA cycle, which included facilitating the conversion of oxaloacetic acid into PEP, and weakening the metabolic flux of PEP to oxaloacetic acid and pyruvate. Moreover, to prevent the retardation of cell growth, the genes of the citric acid transport and TCA cycle were upregulated. Eventually, the appropriate amount of citric acid was added to the medium to enhance the TCA cycle and cell growth. Finally, the titer of tryptophan was further improved to 49 g/L, which is 40% higher than 35 g/L, with a yield of 0.186 g tryptophan/g glucose in a 5 L bioreactor.

## Materials and methods

### Materials

The strains and plasmids used in the experiment are shown in Table 1. The primers used in the experiment are listed in Table 2. Primer STAR HS DNA polymerase was purchased from Takara Bio.Inc. (Dalian, China). 2 × Rapid Taq Mix and ClonExpress ® II One Step Cloning Kit were obtained from VazymeBio. Inc. (Ningjing, China). Oligonucleotides were synthesized by GENEWIZ Bio. Inc. (Suzhou, China).

**Table 1.** Bacterial strains and plasmids used in this study.

Strain/Plasmid	Characteristics	Source
Strains		
* E. coli* DH5α	the cloning host	This lab
* E. coli* W3110	ATCC27325, wild type, starting strain	This lab
* * TRP1	W3110, Δ*lacI*	This study
* * TRP01	TRP1, Δ*tnaA*, Δ*mtr*	This study
* * TRP02	TRP01, P_trc_-trpE*::trpLE	This study
* * TRP03	TRP02, P_trc_-aroG*::tyrR	This study
* * TRP04	TRP03, Δ*pykA*, Δ*ppc*	This study
* * TRP05	TRP04, P*_pck_-pck::ycjV*	This study
* * TRP06	TRP05, P*_citT_-citT::poxB*	This study
* * TRP07	TRP06, P*_lac_-acnBA-icD::yghx*，P*_lac_-pyc::yjiV*	This study
Plasmid		
* * pREDCas9	Spe^r^, Cas9, and λ Red recombinase expression vector	Li et al. (2015)
* * pGRB	Amp^r^, gRNA expression vector	Li et al. (2015)

**Table 2.** Primers.

Primers	Sequences
PGRB-*lacZ*-1	AGTCCTAGGTATAATACTAGTTACGATGTCGCAGAGTATGCGTTTTAGAGCTAGAA TTCTAGCTCTAAAACGCATACTCTGCGACATCGTAACTAGTATTATACCTAGGACT
PGRB-*lacZ*-2	AGTCCTAGGTATAATACTAGTCACTCGCGCTTATCGTGAAGGTTTTAGAGCTAGAA
PGRB-*tnaA*-1	TTCTAGCTCTAAAACCTTCACGATAAGCGCGAGTGACTAGTATTATACCTAGGACT
PGRB-*tnaA*-2	TTCTAGCTCTAAAACACCGTCGCTGCTTGGCGGCGACTAGTATTATACCTAGGACT
PGRB-*mtr*-1	AGTCCTAGGTATAATACTAGTCGCCGCCAAGCAGCGACGGTGTTTTAGAGCTAGAA
PGRB-*mtr*-2	AGTCCTAGGTATAATACTAGTGGTGGCGCACTTCCTGAAACGTTTTAGAGCTAGAA
PGRB-*trpL*-1	TTCTAGCTCTAAAACGTTTCAGGAAGTGCGCCACCACTAGTATTATACCTAGGACT
PGRB-*trpL*-2	AGTCCTAGGTATAATACTAGTCTCGATCTACTCGTGCTAAGGTTTTAGAGCTAGAA
PGRB-*tyrR*-1	TTCTAGCTCTAAAACCTTAGCACGAGTAGATCGAGACTAGTATTATACCTAGGACT
PGRB-*tyrR*-2	AGTCCTAGGTATAATACTAGTACAAAAATCGTTACCACGTTGTTTTAGAGCTAGAA
PGRB-*pykA*-1	TTCTAGCTCTAAAACAACGTGGTAACGATTTTTGTACTAGTATTATACCTAGGACT
PGRB-*pykA*-2	AGTCCTAGGTATAATACTAGTCGTAGTAATGTCAGTATGCTGTTTTAGAGCTAGAA
PGRB-*ppc*-1	TTCTAGCTCTAAAACAGCATACTGACATTACTACGACTAGTATTATACCTAGGACT
PGRB-*ppc*-2	AGTCCTAGGTATAATACTAGTCAAAGCACGCAATATAGCGAGTTTTAGAGCTAGAA
PGRB-*ycjV*-1	TTCTAGCTCTAAAACTCGCTATATTGCGTGCTTTGACTAGTATTATACCTAGGACT
PGRB-*ycjV*-2	AGTCCTAGGTATAATACTAGTTATCGCCAAAACACTCGAATGTTTTAGAGCTAGAA
PGRB-*poxB*-1	TTCTAGCTCTAAAACATTCGAGTGTTTTGGCGATAACTAGTATTATACCTAGGACT
PGRB-*poxB*-2	AGTCCTAGGTATAATACTAGTGGTGCCTGACGACCATAAAAGTTTTAGAGCTAGA
PGRB-*yghX*-1	TCTAGCTCTAAAACTTTTATGGTCGTCAGGCACCACTAGTATTATACCTAGGACT
PGRB-*yghX*-2	AGTCCTAGGTATAATACTAGTATCCCGCATTTCTTAAAGTCGTTTTAGAGCTAGAA
PGRB-*yjiv*-1	TTCTAGCTCTAAAACGACTTTAAGAAATGCGGGATACTAGTATTATACCTAGGACT
PGRB-*yjiv*-2	AGTCCTAGGTATAATACTAGTTGCGCTGGTTGATTTCTTCTGTTTTAGAGCTAGAA
PGRB-Ex1–1	TTCTAGCTCTAAAACAGAAGAAATCAACCAGCGCAACTAGTATTATACCTAGGACT
PGRB-Ex1–2	AGTCCTAGGTATAATACTAGTATGAACATAACTCAATTTGTGTTTTAGAGCTAGAA
PGRB-Ex2–1	TTCTAGCTCTAAAACACAAATTGAGTTATGTTCATACTAGTATTATACCTAGGACT
PGRB-Ex2–2	AAAACCGCCTCGGGCCTGACCT
*LacI*-1	GGAAACCTGTCGTGCCAGCTGGTTTCACATTCACCACCCTGAATT
*LacI*-2	AATTCAGGGTGGTGAATGTGAAACCAGCTGGCACGACAGGTTTCC
*LacI*-3	AGCGAGTAACAACCCGTCGGATTCT
*LacI*4	AGCCAGCGATTGATGGTCTTG
*tnaA*-1	GGTTCGTACGTAAAGGTTAATCCTTAACGGTTCAGGGAGATGTTTAAAGT
*tnaA*-2	ACTTTAAACATCTCCCTGAACCGTTAAGGATTAACCTTTACGTACGAACC
*tnaA*-3	TAATACGACTGGCGGCTAACGAACT
*tnaA*-4	ATATGCAGCTGTACCGCATTGAA
*mtr*-1	AACACCAGAATCAGCGCAATCATCGTGCCATGAGGGCTTCTCTCCAGT
*mtr*-2	ACTGGAGAGAAGCCCTCATGGCACGATGATTGCGCTGATTCTGGTGTT
*mtr*-3	GTTTGCGTTTGCCTCACACTTC
*mtr*-4	AAAGACATCCGCCATTGAACTG
trpE*-1	CGATATCTGCGGATTCCAGCAGCAGCGTTGCCGG
trpE*-2	CCGGCAACGCTGCTGCTGGAATCCGCAGATATCG
trpE*-3	TACGGGCTGGGATTACTCTTTT
trpE*-4	CCGCTCACAATTCCACACATTATACGAGCCGGATGATTAATTGTCAATGTCGATACCCTTTTTACGTGAAC
trpE*-5	TAATGTGTGGAATTGTGAGCGGATAACAATTTCACACAAGGAGATATACCATGCAAACACAAAAACCGACTCTC
trpE*-6	CACCGCTGGCAAGGCTTAGAGT
aroG*-1	GAAGCCGACCGGACAAAAAAGCCCTGATGC
aroG*-2	GCATCAGGGCTTTTTTGTCCGGTCGGCTTC
aroG*-3	CAAACCAGGGTAAAGCGAAGTA
aroG*-4	AGCCGTTTGCGTCTGTTTAAG
aroG*-5	TTGTTATCCGCTCACAATTCCACACATTATACGAGCCGGATGATTAATTGTCAAGGGCACTTCGGCGTAAAGAT
aroG*-6	TGGAATTGTGAGCGGATAACAATTTCACACAAGGAGATATACCATGAATTATCAGAACGACGATTTAC
aroG*-7	GGATTACTATGACACGTCATTCGTTTAAAATGAGAAAG
aroG*-8	AAACGAATGACGTGGTGATGTTGCGATCAACGAT
aroG*-9	TGCACCAGTTCGACCAGATTC
aroG*-10	GATTAGGCTCTTCAGCCGCCGT
*pykA*-1	ACCTTTATCGCGCAGCAGATTAACCTCTGGACATGTAATACTCCGTTGAC
*pykA*-2	GTCAACGGAGTATTACATGTCCAGAGGTTAATCTGCTGCGCGATAAAGGT
*pykA*-3	CTGCATGCGAGAAATGACGGTAT
*pykA*-4	CGCATCTTATCCGACCTACAGTGAC
*ppc*-1	ACTCGGCCTGCAATACGTTCAGCGGAATATTGTTCGTTCATATTACC
*ppc*-2	GGTAATATGAACGAACAATATTCCGCTGAACGTATTGCAGGCCGAGT
*ppc*-3	CCATAGCACCACGCCGATTACT
*ppc*-4	GAATGCGCCGACGATTTTAG
*pck*-1	CACCTTTTGATTCTGGATAACCAACGCCGACAAACACGATGAACTC
*pck*-2	GAGTTCATCGTGTTTGTCGGCGTTGGTTATCCAGAATCAAAAGGTG
pck-3	GGGTCACGTAGATCATGGTGGTTACAGTTTCGGACCAGCCGCTA
*pck*-4	TAGCGGCTGGTCCGAAACTGTAACCACCATGATCTACGTGACCC
*pck*-5	CACCAGCACGGACCACTAACT
*pck*-6	GGCAGGTGTACCTGACTTAGCTTC
*citT*-1	TGAGATTTCGTTCGATACACTCCCTGGTTCTCCATCTCCTGAATGTGATA
*citT*-2	TATCACATTCAGGAGATGGAGAACCAGGGAGTGTATCGAACGAAATCTCA
*citT*-3	CTACTGAGAGGAAATCGCCCATTTAGTTCCACATGGCGAGAATCG
*citT*-4	CGATTCTCGCCATGTGGAACTAAATGGGCGATTTCCTCTCAGTAG
*citT*-5	CTTGGTCGGGTAACGGTATCAC
*citT*-6	GCGCAACGTAGAACAGGAATT
*acnB*-1	TTGTTATCCGCTCACAATTCCACACAACATACGAGCCGGAAGCATAAAGTGTAAAGGAGTAGTAAAGGCGCTTCAATC
*acnB*-2	TGTGGAATTGTGAGCGGATAACAATTTCACACAGGAAACAGCTGTGCTAGAAGAATACCGTAAGCACG
*acnB*-3	TTCTAGCTCTAAAACAGAAGAAATCAACCAGCGCAACTAGTATTATACCTAGGACT TTAAACCGCAGTCTGGAAAATCACC
*acnB*-4	AGTCCTAGGTATAATACTAGTTGCGCTGGTTGATTTCTTCTGTTTTAGAGCTAGAA
*acnB*-5	GAGCAGGTATTTACGTGAACCG
*acnB*-6	GTATTCAGGGCATGGAAAAATGGCT
*acnA*-1	GGTTGACGACATGGTATATCTCCTTTTAAACCGCAGTCTGGAAAATCACC
*acnA*-2	GGTGATTTTCCAGACTGCGGTTTAAAAGGAGATATACCATGTCGTCAACC
*acnA*-3	TTCTAGCTCTAAAACACAAATTGAGTTATGTTCATACTAGTATTATACCTAGGACTTTACTTCAACATATTACGAATGACA
*acnA*-4	AGTCCTAGGTATAATACTAGTATGAACATAACTCAATTTGTGTTTTAGAGCTAGAAGTCATAGTAATCCAGCAACTCTTGTG
*acnA*-5	GAGCAGGTATTTACGTGAACCG
*acnA*-6	AAGACTTTAACTCCTACGGTTCGCG
*icd*-1	TTTACTTTCCATGGTATATCTCCTTTTACTTCAACATATTACGAATGACA
*icd*-2	TGTCATTCGTAATATGTTGAAGTAAAAGGAGATATACCATGGAAAGTAAA
*icd*-3	CACAAGAGTTGCTGGATTACTATGACTTACATGTTTTCGATGATCGCGTCA
*icd*-4	TGACGCGATCATCGAAAACATGTAAGTCATAGTAATCCAGCAACTCTTGTG
*icd*-5	GAGCAGGTATTTACGTGAACCG
*icd*-6	GTGCTGGAGGGATGATTGTTG
*pyc*-1	TTGTTATCCGCTCACAATTCCACACAACATACGAGCCGGAAGCATAAAGTGTAAACGCAGTACTTCCTGCTGGCT
*pyc*-2	TGTGGAATTGTGAGCGGATAACAATTTCACACAGGAAACAGCTGTGTCGACTCACACATCTTCAACGC
*pyc*-3	TTCTAGCTCTAAAACAGAAGAAATCAACCAGCGCAACTAGTATTATACCTAGGACT CACGGTGTTGCGGCCGCGAAGCAGC
*pyc*-4	AGTCCTAGGTATAATACTAGTTGCGCTGGTTGATTTCTTCTGTTTTAGAGCTAGAACGAATTACCGGCGTTCACTATA
*pyc*-5	TCACCTCCACCAGCACATCC
*pyc*-6	GTGGTTCCGACTTTGAAACTGCTGT
*pyc*-7	TATAGTGAACGCCGGTAATTCGTTAGGAAACGACGACGATCAAGTCG
*pyc*-8	CGACTTGATCGTCGTCGTTTCCTAACGAATTACCGGCGTTCACTATA
*pyc*-9	TCACCTCCACCAGCACATCC
*pyc*-10	

### Plasmids, strain, and culture conditions

*E. coli* DH5α was used as a cloning host for plasmid construction; *E. coli* W3110 was used as the starting strain for producing tryptophan. Genetic modification was performed using CRISPR/Cas9 system, and Professor Chen Tao of Tianjin University, China provided plasmids pREDCas9 and pGRB.

The strains obtained in the study were cultured in LB (Luria-Bertani) medium, supplemented with aspirin (final concentration 100 μg/mL), ampicillin (final concentration 50 μg/mL), IPTG (0.1 mM), and arabinose (20 mM) as needed, at temperatures 32°C, 37°C, or 42°C.

### Construction of gRNA plasmids and donor DNA

The two single-stranded DNA were annealed to form a double-stranded DNA, which included the gRNA spacer sequence of the target gene and the homologous sequence of the plasmid pGRB. The double-stranded DNA and linearized pGRB were ligated via homologous recombination using ClonExpress ® II One Step Cloning Kit, and a gRNA expression plasmid was obtained.

The target gene fragment for integration included the upstream and downstream homologous arms (400–600 bp) and the target gene. After purification, the three fragments were overlapped by overlapping PCR, and used for electroporation experiments after recovery by gel.

### Process of target gene integration

The pREDCas9 plasmid was first transformed into CaCl2-treated strain W3110 by chemical transformation. A single transformed colony was cultured overnight at 32°C in LB medium, and transferred to 2XYT medium (1.6% peptone, 1.0% yeast powder, 0.5% NaCl), and incubated at 32°C. At an OD_600_ of approximately 0.1, IPTG was added to induce the expression of the λ Red recombination system. The cells were grown until OD_600_ reached 0.6–0.8 to produce electrocompetent cells. The electroporation system consisted of 100–200 ng of the target gene and 100 ng of the gRNA expression plasmid. The electroporated cells were immediately resuscitated in 1 mL LB for 2 h at 32°C, and 100 μL was streaked on LB medium plates supplemented with ampiciiin and spectinomycin.

For gRNA plasmid curing, positive clones were enriched overnight at 32°C in LB medium with arabinose. For pREDCas9 plasmid curing, positive clones that successfully eliminated plasmid gRNA were grown in LB medium at 42°C overnight.

### Cultivation in shaker flasks

The strains, including all strains obtained in this study, were cultured on agar slant medium at 37°C for at least 12 h. The components of the slant medium (per liter) included glucose 1 g, peptone 10 g, beef extract 5 g, yeast powder 5 g, NaCl 2.5 g, and agar 25 g. The activated strains were transferred to a flask containing 30 mL seed medium, culturing at 37°C with orbital shaking at 200 rpm for 12 h, and then transferred to the fermentation medium with 10% inoculum, and incubation continued for 24 h. The seed medium (per liter) contained glucose 30 g, yeast powder 2 g, KH_2_PO_4_ 2 g, citric acid 2 g, (NH_4_)_2_SO_4_ 2.5 g, MgSO_4_·7H_2_O 1 g, FeSO_4_·7H_2_O 2.8 mg, MnSO_4_·H_2_O 1.2 mg, VH (vitamin H) 0.1 mg, VB_1_ (Vitamin B_1_) 0.5 mg, microelement mixture 1 mL. Fermentation medium (per liter) glucose 10 g, yeast powder 3 g, KH_2_PO_4_ 2.5 g, Citric acid 6 g, (NH_4_)_2_SO_4_ 3 g, MgSO_4_·7H_2_O 2 g, FeSO_4_·7H_2_O 20 mg, MnSO_4_·H_2_O 1.2 mg, VH 0.1 mg, VB_1_ 0.5 mg, microelement mixture 1 mL, 2% phenol red. The fermentation process was maintained at pH 7.0–7.2, using phenol red as the pH indicator.

The composition of microelement mixture includes (g/L): Na_2_MoO_4_·2H_2_O 2, NiCl2·6H_2_O 1.5, CaCl_2_·2H_2_O 5, CuSO_4_·5H_2_O 0.5, Al_2_(SO4)_3_·18H_2_O 1, CoCl_2_·6H_2_O 2, ZnSO_4_·2H_2_O 0.5, H_3_BO_3_ 0.1.

### Cultivation in a bioreactor

After the cells, including strains TRP03, TRP05, and TRP07, were grown on an LB agar slant, they were transferred to a 5 L bioreactor (Baoxing, Shanghai, China) containing 2 L seed medium, and fresh fermentation medium was added to the bioreactor until the OD_600_ of the cells reached 10–14 with 15–20% inoculum, and the fermentation process was started. During the whole fermentation process, the pH was maintained at approximately 7.0 by adding NH_4_OH (25%, v/v), the temperature was maintained at 37°C, and the dissolved oxygen was controlled between 25%–35%. After the glucose was consumed, 80% (m/v) glucose solution was added to maintain the glucose concentration in the fermentation medium at 0.1–5 g/L.

### Substrate and product analysis

Cell growth was monitored by measuring the absorbance at 600 nm (OD_600_). Glucose concentration and glutamate was measured using an SBA-40E biosensor (Institute of Shandong Academy of Sciences, Jinan, Shandong). The concentration of tryptophan in the shaker flask and bioreactor was detected by high performance liquid chromatography (Thermo Scientific UltiMate 3000); Gemini C18 column (Phenomenex, USA) was used as the measuring column. The mobile phase was an acetonitrile-water mixture at a volume ratio 2:98. The flow rate was controlled at 1 mL/min. The detection wavelength and temperature were 278 nm and 40°C, respectively. And acetate was determined using HPLC with an Aminex HPX-87H column (300 mm × 7.8 mm; Bio-Rid, Hercules, CA) at 50°C, 50 mM H_2_SO_4_ as the mobile phase and a flow rate of 0.5 mL/min.

### Data statistics

The dates shown in paper were obtained from a series of experiments with consistent results (at least three repeats per condition).

The formula of standard deviation was as follow

$$S=\sqrt{\frac{1}{N-1}\overset{N}{\underset{i=1}{\sum }}{\left(X_{i}-X\right)}^{2}}$$

S was standard deviation, *N* was sample quantity, *X_i_* was sampling value, $\bar{X}$ was the average of *X_1_, X_2_*, *X_3_* ……. *X_n_*.

The yield (Y) was calculated as quality of tryptophan produced per gram of glucose consumed (g/g glucose).

$$Y=\frac{cv}{M}$$

*c* was defined as the mass concentration of tryptophan, *v* was the volume of the final fermentation broth, and *M* was the total quality of consumed glucose.

The productivity of tryptophan (*Q_c_*) was defined as the tryptophan (g) produced per hour per liter of the fermentation (g/L/h).

$$Q_{c}=\frac{c_{2}-c_{1}}{t_{2}-t_{1}}$$

*c_2_* and *c_1_* were the tryptophan produced (g/L) at the time t_2_ and *t_1_*, respectively.

## Results and discussion

### Construction of tryptophan producing strain TRP03

#### Downregulation of the tryptophan degradation pathway and transport system

**Figure 3.**
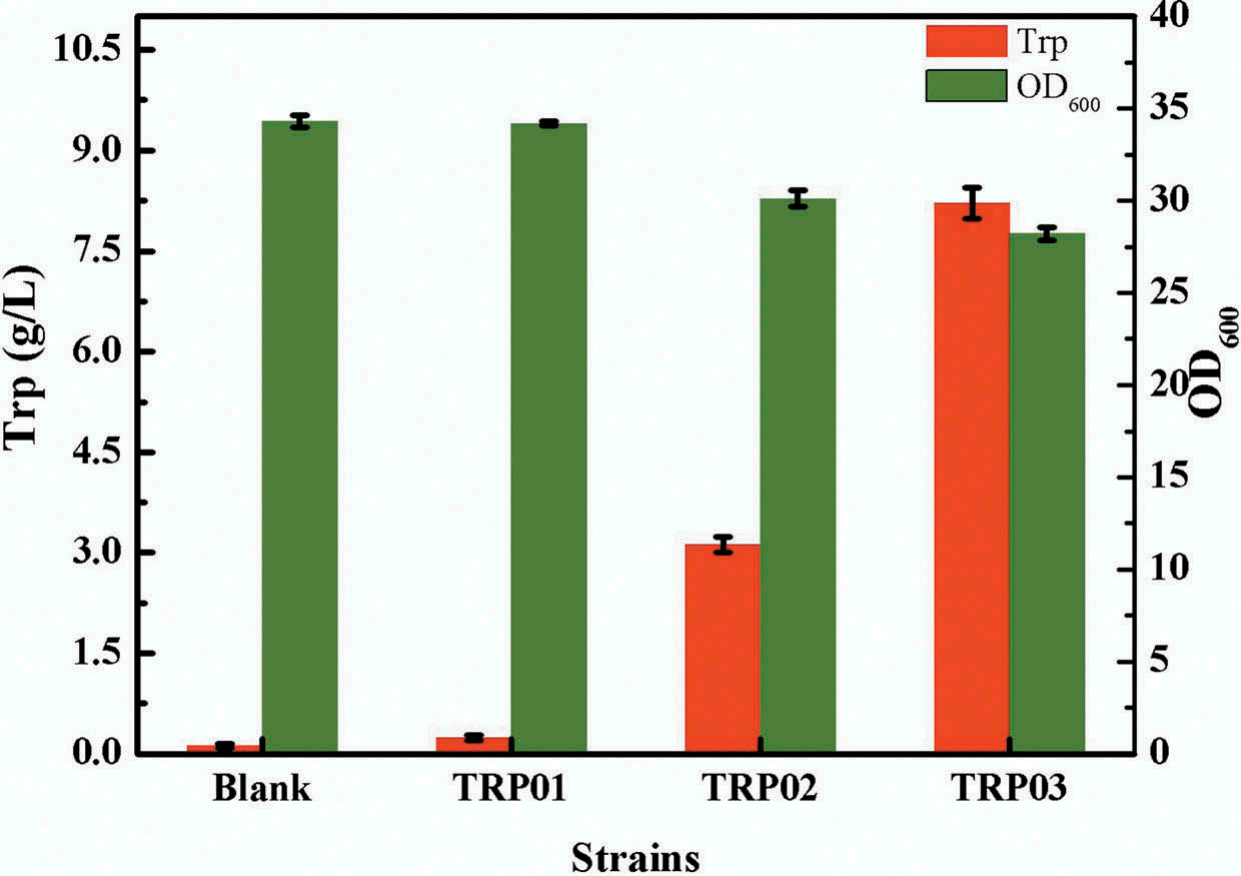
Effect of different modifications on the titer of tryptophan, ‘Blank’ represents strain TRP1.

Mtr, TnaB, and AroP are the three transporters in *Escherichia coli* related to L-tryptophan. Among these, Mtr and TnaB exhibit specific affinity to L-tryptophan. The affinity of Mtr to tryptophan is higher than that of TnaB. AroP is a transporter that can absorb three aromatic amino acids [35]. In this study, TRP1 was chosen as the starting strain, the genome of which was deleted from *lacI*, and the promoters of trc and lac could function normally without an inducer. First, *tnaA*, a member of the *tna* operon [36], and *mtr* were knocked out to block the tryptophan degradation pathway and translocate tryptophan into cells; consequently, the TRP01 strain was obtained. Because the tryptophan synthesis pathway was not upregulated, the decrease of tryptophan degradation and transport could not accumulate tryptophan obviously. In addition, to avoid making drastic changes to the cellular metabolism, other transport systems were not modified. The amount of tryptophan accumulated by the TRP01 strain relative to that by the TRP1 is shown in Figure 3.

#### Enhancement of the branch acid synthesis pathway

The branching pathway of L-tryptophan is one of the three branches of *E. coli*, which synthesize aromatic amino acids. The branching pathway of L-tryptophan mainly relies on the enzyme system encoded by the tryptophan operon [37]. Therefore, the intensity of expression of the tryptophan operon in the target strain directly determines its ability to produce L-tryptophan [38]. Anthranilate synthase is a key enzyme for tryptophan biosynthesis, and the activity of the enzyme is affected by L-tryptophan. P*_trc_-trpE* (S40F) was integrated into the *trpLE* locus, and the promoter of the tryptophan operon was replaced with the trc promoter and the leading peptide gene coded by *trpL* was deleted, which affected tryptophan operon activity; the resulting strain was named TRP02. After shaking flask for 24 h, the titer of tryptophan, which reached approximately 3 g/L, was greatly improved compared to the TRP01 strain with a slight decrease in bacterial growth; OD_600_ dropped from 35.2 to 30.4 (Figure 3).

#### Upregulation of the shikimic acid pathway

In addition to the choline acid synthesis pathway, the shikimic acid pathway is important for tryptophan accumulation [39]. The activity of 3-deoxy-d-arabeptone-7-phosphate synthase directly affects the flow of PEP to the shikimic acid pathway, which determines the final accumulation of tryptophan. In this experiment, P*_trc_-aroG* (S211F) was integrated into the *tyrR* locus to enhance the shikimic acid pathway and relieve the transcriptional repression of the key enzyme by TyrR protein to obtain the strain TRP03. After 24 h of the final fermentation step, 8 g/L of tryptophan was accumulated, showing a significantly high production capacity, which had improved 170.96% compared to that of the strain of TPR02, the genetic modification did not affect cell growth (Figure 3).

To further improve the production of tryptophan, TRP03 was used as a chassis strain, and the central metabolic pathway of this strain was modified to redistribute the metabolic flux distribution of the PEP and pyruvic acid.

### Modification of the EMP pathway

#### Downregulation of the PEP metabolic pathway

**Figure 4.**
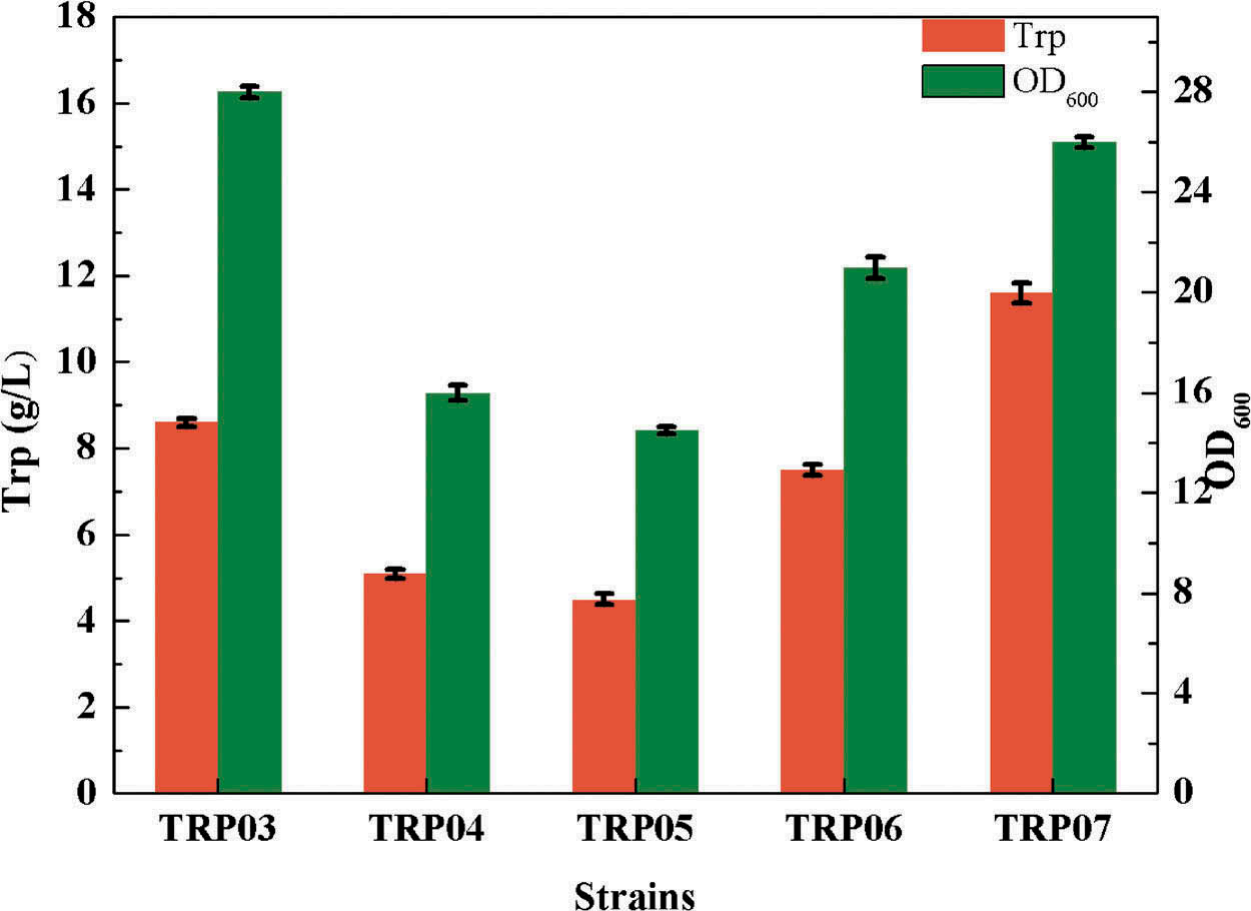
Effect of different modifications of central metabolic pathway on the titer of tryptophan.

PEP is not the only precursor for the synthesis of tryptophan; PEP enters the TCA cycle through pyruvic acid, which is the main route of PEP, to provide energy for cells. There are two pyruvate kinases in *E. coli*, namely, pyruvate kinase I and pyruvate kinase II [40]. In addition, PEP can synthesize oxaloacetic acid via phosphoenolpyruvate carboxykinase [41] (coding gene *ppc*), which provides a steady stream of intermediate metabolites for the TCA cycle. To attenuate the metabolic flux of PEP to pyruvate and oxaloacetic acid, *pykA*, encoding pyruvate kinase II, and *ppc* were knocked out to obtain the strain TRP04. The deletion of *ppc* and *pykA* greatly affected the cell growth (Figure 4), but the yield of tryptophan was increased. The possible reason might be that the modification of the *ppc* and *pykA* affected the metabolism of oxaloacetic acid and pyruvate into the TCA cycle. The flow distribution blocked the TCA cycle, thereby inhibiting cell growth. Consequently, the titer of tryptophan was 4 g/L, reduced by 45.21% compared with TRP03, and OD_600_ of the bacterial strain TRP04 was only 9.1.

#### Enhancement of the PEP synthesis pathway

For further strengthen the supply of PEP, we also enhance the PEP synthesis pathway. In *E. coli*, pyruvate and oxaloacetic acid can synthesize PEP by different enzymes, and phosphoenolpyruvate kinase (coding gene *pck*) [42] can catalyze the synthesis of PEP from oxaloacetic acid. Similarly, phosphoenolpyruvate synthase (coding gene *pps*) [43] can act on pyruvate to translate into PEP. To further increase the supply of PEP, the pathway of PEP by oxaloacetic acid was enhanced to obtain the strain TRP05. The results were as expected. The enhancement of the gene *pck* weakened the TCA cycle, eventually leading to a decrease in cell growth and tryptophan production. OD_600_ was 8.4 and the titer of TRP was 9 g/L (Figure 4).

### Enhancement of the TCA cycle

#### Enhancement of citric acid transport

The TCA cycle, also known as the citric acid cycle, is a key metabolic pathway in cells, and provides energy for cell metabolism [44]. In this study, due to the modification of the EMP pathway, the propagation of TRP05 was challenging; hence, we were unable to estimate the capacity of this strain to accumulate tryptophan. To solve this problem, the gene *citT* was up-regulated, which can transport citric acid into intracellular. Considering that excessive enhancement of the transporter also affects the cells, the promoter of *citT* itself was chosen to regulate the transcription of the gene *citT*, and the strain TRP06 was obtained. During the fermentation experiments, approximately 6 g/L citric acid was added to enhance the TCA cycle. Commendably, the addition of citric acid could solve the problem of cell growth to some extent. After shaking for 24 h, the OD_600_ of bacteria could be increased to 22, and the accumulation of tryptophan was improved considerably to 7 g/L from 4 g/L (Figure 4).

#### Enhancement of the TCA cycle

To accelerate the transport of citric acid into the TCA cycle, we further enhanced the metabolic pathway of citric acid in the TCA cycle. For making the genes regulated by the same promoter, we used the gene large fragment integration method [45]. to express the coding genes *acnBA* and *icd* in tandem regulated by P*_acnB_* the operation flow chart  was showed in Figure 2. This strategy could accelerate the incorporation of citric acid into the TCA to improve their growth. Simultaneously, in order to replenish oxaloacetic acid from pyruvate, pyruvate carboxylase from *Corynebacterium glutamicum* for heterologous expression was used to obtain the strain TRP07. Although the enhancement of the synthesis pathway of PEP through oxaloacetic acid competed for some carbon flow because of the upregulation of *pck*, there was still some flow of oxaloacetic acid into TCA cycle; even trace amounts of oxaloacetic acid could partially recharge the TCA cycle. In the results, as shown in Figure 4, the OD_600_ of the strain TRP07 could reach to approximately 26, the cell growth was restored, and the accumulation of tryptophan increased to 12 g/L.

### Fermentation experiment in a biosensor

**Figure 5.**
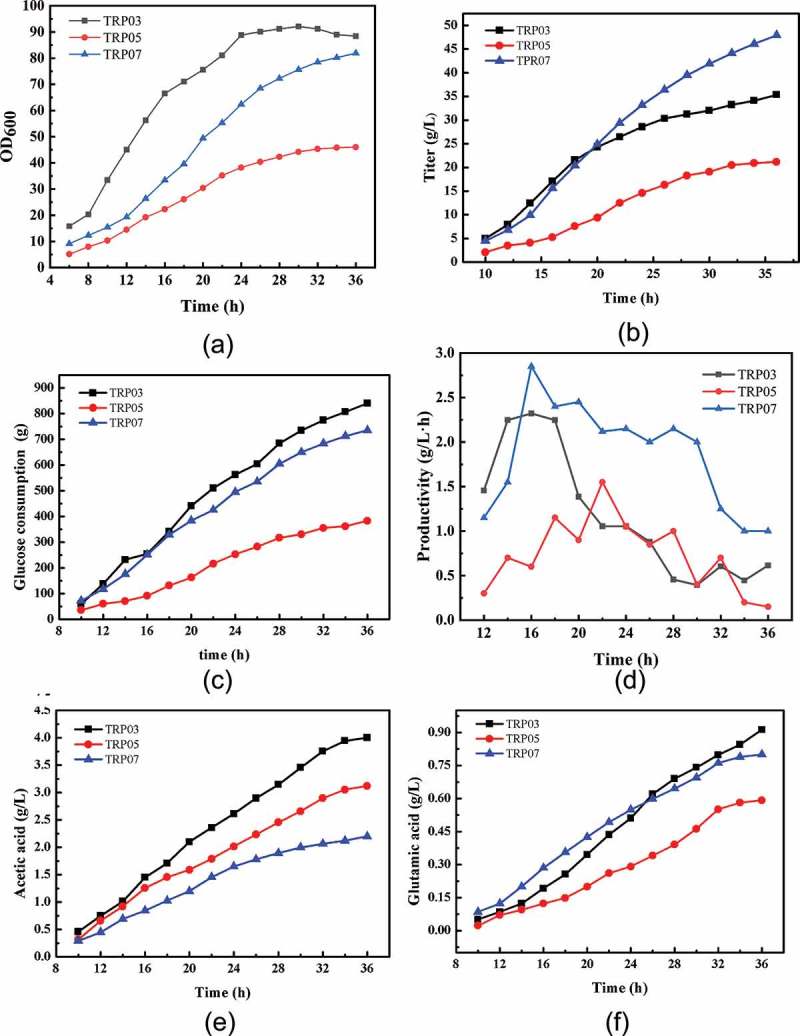
Fed-batch fermentation profiles of the engineered strains TRP03, TRP05, and TRP07 in a 5 L bioreactor. Cell growth (A), tryptophan titer (B), glucose concentration, (C), productivity (D), acetic acid (E), and glutamate (F)are shown. The data shown were chosen as a typical result from a series of experiments.

To comprehensively evaluate the overall production performance of the *E. coli* strains including TRP03, TRP05, and TRP07, fed-batch fermentation was carried out in a 5 L bioreactor. Glucose (80%) was fed into the bioreactor when the glucose concentration was lower than 5 g/L. Citric acid was subsequently fed into the bioreactor at a 2–5 g/L rate for TRP07 after fermentation for 10 h. The details of the fermentation process are illustrated in Figure 5. The chassis strain TRP03 grew faster at the start of the fermentation process, and OD_600_ reached 90 at 24 h, but after 24 h, the cells no longer grew, leading to a decrease in tryptophan accumulation. In addition, the strain TRP05 could not grow normally because of the lack of intermediate metabolites in the TCA cycle, and tryptophan accumulation and glucose consumption rate slowed. The cells grew slowly and the titer of tryptophan was lower than that of TRP03 at the beginning of fermentation, but showed higher growth and acid production ability after 14 h. In the case of TRP07, OD_600_ reached to 81 at 36 h and the yield of TRP07 was 0.186 g/g glucose, which was increased to 28.75% compared with that of TRP03. Cells still showed high growth activity up to 36 h, and tryptophan production capacity increased, finally tryptophan could accumulate to 49 g/L. The results shown that the problems of cell growth were successfully solved by the addition of citric acid and TCA intermediate metabolite replenishment. Furthermore, the productivity of TRP07 was increased to 2.8 g/L/h, and by 21.74% compared to that by TRP03. Simultaneously, acetic acid and glutamate were analyzed as the by-products (Figure 5). Compared to the TRP03 strain, acetic acid in TRP07 strain did not accumulate in large quantities (approximately 2.2 g/L). The main reasons may be decreased pyruvic acid accumulation due to *pykA* deletion, and the introduction of the *pyc* from *Corynebacterium glutamicum*, thereby the flow of pyruvic enters the acetic acid was reduced. Acetic acid accumulation by strain TRP05 was higher than that by strain TRP07, and lower than that by strain TRP03, which was as expected. As for glutamate accumulation, because of the enhancement of TCA, α-ketoglutarate increased and flowed to the glutamate pathway, the accumulation of glutamate was higher in TRP07 than that in TRP03 at the end of 24 h. However, after 24 h, the accumulation of glutamic acid in strain TRP03 was higher than that in strain TRP07. This may be due to a decrease in cell growth and tryptophan production. Furthermore, glutamate accumulation of strain TRP05 was lower than that of strain TRP03 and strain TRP07, which may be caused by the weakening of TCA cycle.

However, complex synthesis pathways and regulatory mechanisms of tryptophan always lead to a low production yield. PEP, one of the precursors required for tryptophan biosynthesis, also enters the TCA cycle through pyruvate to provide energy for cell growth, so a large number of PEP enters the tryptophan biosynthesis pathway will influence cell growth enormously due to insufficient energy supply. Therefore, the ideal dynamic balance between cell growth and tryptophan production will be helpful for tryptophan production. In this study, the synthesis pathway of PEP to tryptophan was strengthened, the reactions of PEP to TCA were weakened, and citric acid transport was enhanced. In this way, we expect to improve the yield of tryptophan. The final strain TRP07 could produce tryptophan 49 g/L and the yield was 0.186 g/g glucose, representing the highest reported lever (Table 3). However, the effect of this study is far from the theoretical maximum, which means that there is still much room to improve the yield of tryptophan by balancing the ratio of biomass formation and tryptophan production.

**Table 3.** Overview on tryptophan production with different strains.

Microorganism	Trp titer (g/L)	Yield (g/g glucose)	Reference
*E. coli* S028	30–40	0.15	Lin Chen et al. (2016)
*E. coli* S092/pAnTrpC	29	0.18	Lin Chen et al. (2018)
*E. coli* FB-04(pta1)	44	0.13	Lina Liu et al. (2016)
*E. coli* TRP07	49	0.186	This study

## Conclusion

In this study, we have reported the construction of an engineered *E. coli* strain capable of efficiently producing tryptophan by modifying the EMP and TCA cycle. The flux of PEP to the tryptophan synthesis pathway was enhanced by modifying the metabolism of PEP, and the problem of cell growth was solved by enhance the citric acid transport and TCA cycle. TRP07 could accumulate 49 g/L tryptophan at 36 h in a 5 L bioreactor. The yield was 0.186 g tryptophan/g glucose, and the productivity of TRP07 was up to 2.8 g/L/h. The strategy revealed in this paper may provide further insight into for improvement in tryptophan production, and the yield from glucose.
